# Enhanced Electromechanical Response in PVDF-BNBT Composite Nanofibers for Flexible Sensor Applications

**DOI:** 10.3390/ma15051769

**Published:** 2022-02-26

**Authors:** Chung Ming Leung, Xiaoqiu Chen, Tao Wang, Yanxue Tang, Zhihua Duan, Xiangyong Zhao, Helezi Zhou, Feifei Wang

**Affiliations:** 1 School of Mechanical Engineering and Automation, Harbin Institute of Technology (Shenzhen), Shenzhen 518055, China; 2 Key Laboratory of Optoelectronic Material and Device, Department of Physics, Shanghai Normal University, Shanghai 200234, China; cxq991205@163.com (X.C.); twang@shnu.edu.cn (T.W.); yanxuetang@shnu.edu.cn (Y.T.); zhihuaduan@shnu.edu.cn (Z.D.); xyzhao@shnu.edu.cn (X.Z.); 3 State Key Laboratory of Materials Processing and Die & Mould Technology, School of Materials Science and Engineering, Huazhong University of Science and Technology, Wuhan 430074, China

**Keywords:** PVDF, BNBT, composite nanofiber, flexible device

## Abstract

Wearable energy harvesters and sensors have recently attracted significant attention with the rapid development of artificial intelligence and the Internet of Things (IoT). Compared to high-output bulk materials, these wearable devices are mainly fabricated by thin-film-based materials that limit their application. Therefore, the enhancement of output voltage and power for these devices has recently become an urgent topic. In this paper, the lead-free bismuth titanate-barium titanate (0.93(Na_0.5_Bi_0.5_)TiO_3_-0.07BaTiO_3_(BNBT)) nanoparticles and nanofibers were embedded into the PVDF nanofibers. They produced high inorganic electrical voltage coefficients, high electromechanical coupling coefficients, and environmentally friendly properties that enhance the electromechanical performance of pure PVDF nanofibers, and they are all the critical requirements for modern flexible pressure sensors. In detail, PVDF and PVDF-based composites nanofibers were prepared by electrospinning, and different flexible sandwich composite devices were fabricated by the PDMS encapsulation method. As a result, the six-time enhancement maximum output voltage was obtained in a PVDF-BNBT (fiber)-based composite sensor compared to the pure PVDF one. Our results indicate that the output voltage of the pressure sensors has been significantly enhanced, and the development gate is enabled by analyzing the related physical process and influence mechanism.

## 1. Introduction

In recent years, microelectromechanical systems (MEMS) devices, energy harvesters, and flexible sensors have received rapid development based on the extensive growth and enhanced performance of the piezoelectric nanostructures [1,2,3,4,5]. Among them, flexible sensors based on the piezoelectric nano-materials and their direct effect have the advantages of simple structure, long life, low power consumption, high sensitivity, and are conducive to integration and miniaturization, which has raised a lot of research efforts. Compared to single crystals and ceramics, piezoelectric polymers represented by polyvinylidene fluoride (PVDF) have higher flexibility, lower density, and relatively high piezoelectric voltage constant that become preferred candidates for making flexible pressure sensors [6,7]. In addition, these kinds of piezoelectric polymers have the advantages of low acoustic impedance, making them easier to use in liquid environments such as water, human tissue, other organic liquid, etc.

Nowadays, polymer materials, especially PVDF and PVDF copolymers with high sensitivity and deformability, are considered as the most promising materials, which can meet the requirements of the flexibility characteristics and the dynamic tactile sensing of wearable electronic devices products [8,9,10]. In 2015, Won et al. prepared power generators based on the copolymer of PVDF thin film-based and paper substrates, which can generate maximum voltages of 0.4 and 1.5 V at bending frequencies of 0.25 and 2 Hz under open-circuit conditions, respectively [11]. However, the preparation process of PVDF piezoelectric organic film is very complicated. It can only obtain piezoelectricity through high-voltage polarization and mechanical stretching, resulting in the problems of significant energy consumption and high production costs.

Compared with bulk film materials, one-dimensional ferroelectric materials with ultra-high specific surface area and excellent piezoelectric properties showed good application prospects in piezoelectric devices [12,13]. To simplify the fabrication process of one-dimensional PVDF nanofibers, researchers proposed the electrospinning method that can enhance output performance. In the 2010s, Wang and Lin et al. demonstrated a high output power (voltage and current) of the piezoelectric generator fabricated by PVDF nanofibers with an electrospinning method [9,13]. Under an in-situ high-voltage electric field during the preparation process, the PVDF nanofibers exhibited stronger piezoelectric properties without the additional fabrication steps of secondary polarization and mechanical stretching [13,14,15,16,17,18,19]. Regarding the relatively low piezoelectric strain constant (*d*) of PVDF, there were still many improvements for developing a commence piezoelectric PVDF device. In addition, research indicated that doping was an effective way to improve the piezoelectric properties of PVDF, which was critical to further enhance the piezoelectric sensor performance [18,20]. Yu et al. increased the surface charge density of PVDF nanofibers by adding 5 wt% of multi-walled carbon nanotubes (MWCNT), and the output voltage was increased to 6 V and was a 200% enhancement compared with pure PVDF devices [17]. Chen et al. proposed the use of piezoelectric-enhanced poly(vinylidene fluoride–trifluoroethylene)/barium titanate (P(VDF-TrFE)/BaTiO_3_) nanocomposite to prepare high-performance flexible piezoelectric nanogenerators, which produced a higher maximum output voltage of 13.2 V [18].

Considering the perovskite inorganic piezoelectric materials having higher piezoelectric and dielectric constants, such as barium titanate (BTO), lead zirconate titanate (PZT), lead magnesium niobate-lead titanate (PMN-PT) [21,22], etc., it can be expected to obtain good elasticity and improve device performance if the high-voltage electrical properties of piezoelectric materials can be combined with the flexible characteristics of PVDF. In this paper, the morphotropic phase boundary composition bismuth sodium titanate-barium titanate (BNBT) was chosen as the core materials for embedding into the PVDF nanofibers and extended the fabrication for the flexible pressure sensors. BNBT is one of the best lead-free piezoelectric materials that received widespread attention and have a high coupling performance of *d*_33_ ~500 pC/N [23]. Therefore, BNBT particles and nanofibers were chosen to improve the output performance of PVDF nanofibers and maintain the soft and flexible properties of the entire composite. On this basis, these proposed composites were further developed in pressure sensors that demonstrated a significant performance improvement compared to pure PVDF devices.

## 2. Structure and Experiments

### 2.1. BNBT Particles and BNBT Nanofibers Fabrication

The BNBT particles and BNBT nanofibers were prepared based on a sol-gel process [24]. For the BNBT particles fabrication, the starting materials, barium acetate, bismuth acetate, sodium acetate, and tetrabutyl titanium, were used to prepare the NBT-0.07BT precursor solution. During the preparation, acetic acid and 2-methoxyethanol with a volume ratio of 1:1 were chosen as solvent. A 5% excess amount of bismuth acetate was added to compensate for possible bismuth loss during high-temperature annealing, and tetrabutyl titanium was first dissolved in acetylacetone to prevent the hydrolysis in air. The final solution was stirred at room temperature for 24 h. After the evaporation of the organic solvent at a high temperature, the BNBT particles can be obtained. For the BNBT nanofibers, an electrospinning technique was further adopted. After getting the BNBT precursor solution, an ethanol solution containing polyvinyl pyrrolidone (PVP, MW = 630,000) was added to the previous precursor solution and stirred to form a homogeneous solution. Then, the composite NBT-0.07BT/PVP solution was loaded into a plastic syringe equipped with a needle and then electrospun using a spinning system. Electrospinning was conducted at 25 kV, 15 cm spacing between needle tip and collector, and a feed rate of 1 mL/h. The as-spun nanofibers were collected on the aluminum foil, accompanied by solvent evaporation. The as-spun fibers were then dried at 90 °C for 4 h and then annealed at 750 °C for 2 h in the air to crystallize.

### 2.2. PVDF Nanofibers and PVDF Composite Nanofibers Incorporated with BNBT Particles and BNBT Nanofibers

For the PVDF spinning solution, the raw PVDF powder was dissolved in the N, N-Dimethylformamide (DMF, Sinopharm Chemical Reagent Corporation, Shanghai, China), and acetone (3/2 *w*/*w*) mixture at a PVDF powder/solvent weight ratio of 1:9. Then, the electrospinning was conducted at 25 kV, 15 cm spacing between needle tip and collector, and a feed rate of 1.5 mL/h. The as-spun nanofibers were dried at 80 °C for 4 h. For the PVDF-BNBT (particle) and PVDF-BNBT (nanofiber) composite nanofibers fabrication, before the electrospinning process, the BNBT particles and BNBT nanofiber (30 wt% of the PVDF powder weight) were first dispersed into the PVDF spinning solution. Then, the electrospinning was conducted under the same conditions as the pure PVDF.

### 2.3. Morphology and Structure Characterization

The crystal structure of the thin film was conducted by X-ray diffraction (XRD) (Bruker D8 focus, Bruker AXS, Karlsruhe, Germany) under Cu Kα radiation. A field emission scanning electron microscope (FESEM, S-4800, Hitachi, Japan) was used to characterize the fiber samples and to determine the diameters of the fibers. The secondary electron image mode was adopted. Both the as-spun nanofibers and annealed samples were coated with gold. Fourier transform infrared (FTIR) spectra of the fibers were measured using a Thermo fisher Nicolet iS10 system (Waltham, MA, USA).

## 3. Results and Discussion

**Figure 1:**
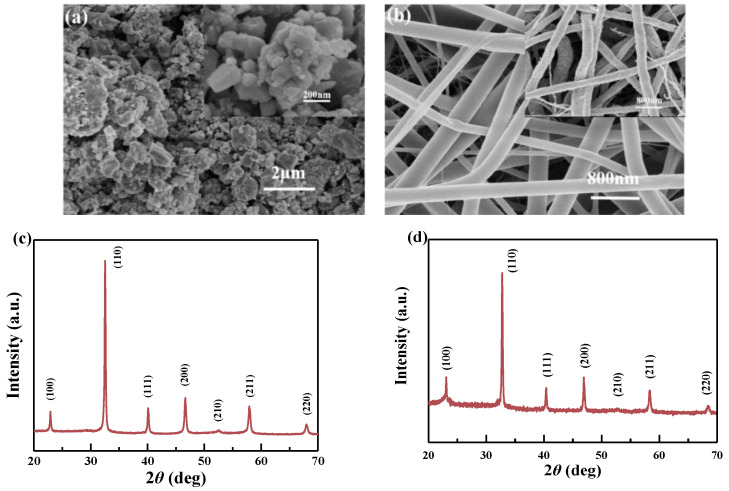
(**a**) BNBT particles topography (inset is partial magnification), (**b**) morphology of as-spun BNBT nanofibers and BNBT nanofibers annealed at 750 °C (inset), (**c**) XRD of BNBT particles, and (**d**) XRD of BNBT nanofibers annealed at 750 °C.

The BNBT particles were obtained from BNBT precursor solution, dried in an oven at about 80 °C to volatilize the solution method, and then annealed at 750 °C [25]. Figure 1a shows the topography of BNBT particles, and the inset is the partial magnification. It can be found that the particle had large specific surface areas, so they were more accessible to agglomerate together, and the single-particle size was ~100 nm or less after annealing. On the other hand, Figure 1b shows the morphology of as-spun BNBT nanofibers (before embedded with PVDF nanofibers), and the insert is as-spun BNBT nanofibers annealed at 750 °C [26]. It can be seen that the surface of the nanofibers was smooth without any adhesion, and the diameter of the nanofibers was ~300–400 nm. However, after BNBT and nanofibers were annealed at 750 °C, it can be found that the surface of annealed nanofibers was rough, the crystal gains increased, and the diameter of nanofiber reduced to 200–300 nm. These findings may result from the surface volatilization of the organic matter on the fiber’s surface and the crystallization as the temperature rises [27,28]. Figure 1c,d show the XRD of BNBT particles and BNBT nanofibers annealed at 750 °C. It can be found that pure perovskite structure and no impurities were found in both materials from the XRD, indicating that the prepared BNBT particles and BNBT fibers were ready to be incorporated with PVDF nanofibers in the next fabrication steps.

**Figure 2:**
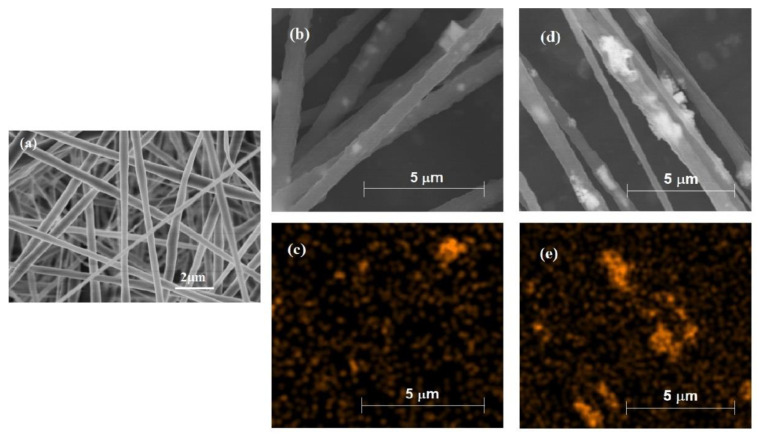
Morphologies of (**a**) pure PVDF nanofibers, (**b**) PVDF-BNBT (particle) composite nanofibers, (**c**) EDS image of PVDF-BNBT (particle) composite nanofibers (the distribution of element Ti), (**d**) PVDF-BNBT (fiber) composite nanofibers, and (**e**) EDS image of PVDF-BNBT (fiber) composite nanofibers (the distribution of element Ti).

Figure 2 shows morphologies of (a) pure PVDF nanofibers, (b) PVDF-BNBT (particle) composite nanofibers, (c) EDS image of PVDF-BNBT (particle) composite nanofibers (the distribution of element Ti), (d) PVDF-BNBT (fiber) composite nanofibers, and (e) EDS image of PVDF-BNBT (fiber) composite nanofibers (the distribution of element Ti). In Figure 2a, it is noted that the surface of pure PVDF nanofibers was smooth and slender that was good for bonding with other particles/fibers. In detail, after PVDF nanofibers were compounded with BNBT particles and BNBT fibers, the diameter and surface morphology of PVDF nanofibers were essentially remained the same, and the diameter of all nanofibers was found at a similar size of ~500 nm. On the other hand, in Figure 2b,c, it is found that there were some particles on the surface of PVDF nanofibers due to the incomplete dispersed, and these particles made rough of the surface while they were bonded with PVDF nanofibers. Figure 2d,e show the surface structure of PVDF-BNBT (fiber) composite nanofiber. It is shown that longer BNBT was found in the EDS image (Figure 2e) of PVDF-BNBT (fiber) nanofibers compared to another one composited with BNBT particles (Figure 2c). It is because annealed BNBT fiber was dispersed into PVDF through ultrasonic vibration. The BNBT long-fiber was broken into short-fiber, and this BNBT short-fiber was bigger than the particle one, making the surface roughness of PVDF-BNBT (fiber) more significant.

**Figure 3:**
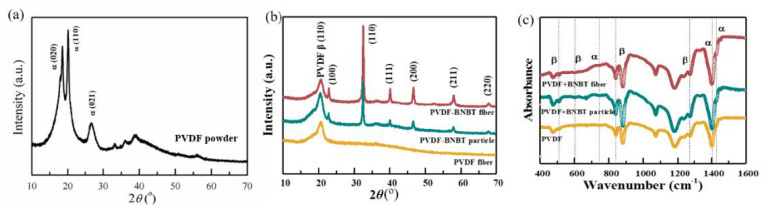
(**a**) XRD of the raw PVDF powder, (**b**) XRD, and (**c**) FTIR of PVDF nanofibers, PVDF-BNBT (particle) composite nanofibers, and PVDF-BNBT (fiber) composite nanofiber.

Figure 3 shows the XRD and FTIR results of PVDF nanofibers, PVDF-BNBT (particle) composite nanofibers, and PVDF-BNBT (fiber) composite nanofibers. In the detail of XRD plots, both PVDF and BNBT characteristic peaks were detected in composites. These results proved that both BNBT particles and BNBT fibers were incorporated into PVDF nanofibers. In addition, we found that the perovskite diffraction peaks of PVDF-BNBT (particle) and PVDF-BNBT (fiber) were sharp, which indicated the BNBT had well crystallization under the high-temperature calcination. From the FTIR spectrum, it was found that the piezoelectric *β* phase can be detected after combining BNBT particles and BNBT nanofibers.

**Figure 4:**
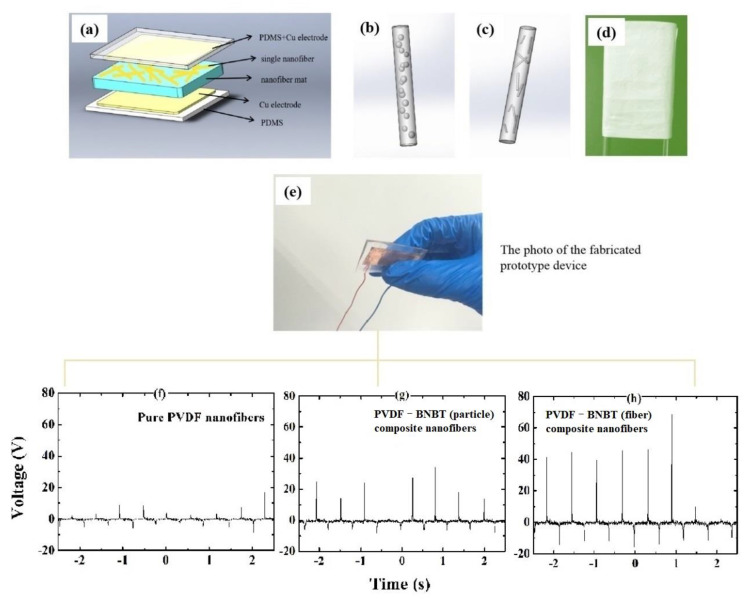
(**a**) Schematic diagram of the device structure, (**b**,**c**) schematic diagram of single PVDF-BNBT (particle) and PVDF-BNBT (fiber) composite nanofiber, (**d**) photograph of a nanofiber mat, (**e**) photograph of the prototype device and voltage outputs of each device based on (**f**) pure PVDF nanofibers with a maximum output voltage of 10 V (RMS output voltage of 1.10 V), (**g**) PVDF-BNBT (particle) composite nanofibers with a maximum output voltage of 30 V (RMS voltage of 2.00 V), and (**h**) PVDF-BNBT (fiber) composite nanofibers with a maximum output voltage of 60 V (RMS output voltage of 3.57 V). All mechanical inputs were applied at the frequency of 1.65 Hz.

Furthermore, to give an insight into the electromechanical performance of different composite nanofibers, three flexible pressure sensors based on PVDF nanofibers, PVDF-BNBT (particle) composite nanofibers, and PVDF-BNBT (fiber) composite nanofibers, respectively, were fabricated as sandwich structures, as shown in Figure 4a–e. In detail, these three nanofibers were encapsulated by the PDMS matrix and formed as nanofiber mats. Then, these nanofiber mats were collected using an aluminum foil and dried at 80 °C for 4 h, and were deposited with the electrode on the top and bottom surface to form the sandwich structure. The size of the fiber mat was 2 × 3 cm^2^, and the thickness was 20 μm. Under the same pressing and releasing process provided by the manual test stand force testing crimper (the applied mass is 100 g), the output signals from each sensor were directly measured by oscilloscope and shown in Figure 4f–h. In detail, we can find that the pure nanofiber-based PVDF pressure sensor produced a maximum output voltage of about 10 V (RMS output voltage of 1.1 V) (Figure 4f). In contrast, under the same excitation conditions, the maximum output voltages of the PVDF-BNBT (particle) nanofiber-based and PVDF-BNBT (fiber) nanofiber-based pressure sensors were 30 V (RMS output voltage of 2 V) (Figure 4g) and 60 V (RMS output voltage of 3.57 V) (Figure 4h), respectively. These results indicated that adding BNBT has effectively enhanced the output voltage of proposed pressure sensors. We considered that the incorporated BNBT is a ferroelectric material with strong spontaneous polarization (above 30 μC/cm^2^) and can create a local electric field. This electric field will induce the nearby PVDF molecule bond mechanical stretching, thereby improving the piezoelectric phase content of the PVDF, accompanying the improvement of the piezoelectric performance. On the other hand, it is found that the PVDF-BNBT (fiber) nanofiber-based sensor produced a better performance than another PVDF-BNBT (particle) nanofiber-based sensor. This result may be due to a larger and longer size of BNBT fibers attached to the surface of PVDF nanofiber, resulting in better local polarization stretching and resulting stress of PVDF-BNBT (fiber) composite nanofiber. Therefore, enhanced piezoelectric and electromechanical responses were obtained.

## 4. Conclusions

In summary, PVDF-BNBT composite nanofibers were investigated, and it was found that the three nanofibers (PVDF, PVDF-BNBT (particle), and PVDF-BNBT (fiber)) had similar diameter, which indicated the embedded process did not change the physical dimension of the nanofiber. An advantage of the enhanced direct piezoelectric effect of the PVDF-BNBT (fiber) composite nanofibers was demonstrated in the application of piezoelectric pressure sensors. In application, three flexible sensors can produce the RMS output voltage of 1.1 V (pure PVDF nanofibers), 2 V (PVDF-BNBT (particle) composite nanofibers), and 3.57 V (PVDF-BNBT (fiber) composite nanofibers), respectively, under the same vibration excitation. It is found that the highest output voltage was obtained in PVDF-BNBT (fiber) composite nanofiber-based piezoelectric sensor and received a giant peak voltage of 60 V, indicating the benefit of adding BNBT short-fiber into the PVDF devices. Besides, the enhanced output for the BNBT particles and fibers was briefly discussed. This work potentially gives a reference and opens a gate for the piezoelectric composite fibers in designing future sensors.

## Data Availability

All the data and results supporting this research paper are already presented within this publication.
